# Bacillus Calmette-Guérin Instillations May Mimic Prostate Cancer on Multiparametric Magnetic Resonance Imaging

**DOI:** 10.7759/cureus.69890

**Published:** 2024-09-21

**Authors:** João Guerra, Joao M Pina, Vanessa Andrade, Miguel Lança, Luís Campos Pinheiro

**Affiliations:** 1 Urology, Centro Hospitalar Universitário de Lisboa Central, Lisboa, PRT

**Keywords:** bcg therapy, granulomatous prostatitis, multiparametric magnetic resonance imaging, prostate, prostate cancer

## Abstract

Granulomatous prostatitis (GP) is a rare and benign inflammatory condition of the prostate, often mimicking prostate cancer (PCa) in clinical and radiological evaluations. This study examines the characteristics and diagnostic challenges of GP in a cohort of 12 patients who received Bacillus Calmette-Guérin (BCG) therapy following treatment for non-muscle-invasive bladder urothelial carcinoma. In this case series, we analyze their clinical presentations, MRI findings, and histopathological results.

Patients presented with elevated PSA levels and firm or nodular prostates on digital rectal examination, complicating the differentiation from PCa. Multiparametric MRI showed lesions with hypointensity on T2-weighted images, hypersignal on diffusion-weighted imaging and hyposignal on the apparent diffusion coefficient map, further mimicking malignancy. Histopathological examination remains the gold standard for diagnosing GP, distinguishing it from PCa through the identification of granulomas and associated inflammatory cells.

This study underscores the importance of awareness and accurate diagnosis of GP to avoid unnecessary biopsies and treatments, highlighting the need for a multidisciplinary approach combining clinical, imaging, and pathological data.

## Introduction

Although intravesical administration of Bacillus Calmette-Guérin (BCG) is part of the treatment of high- and intermediate-risk non-muscle-invasive bladder urothelial carcinomas as it reduces the risk of recurrence, it is not free from side effects. [1] These include granulomatous prostatitis (GP), a condition caused by urine contaminated by BCG, usually asymptomatic, with local or systemic reactions in only 1-3% of patients. On digital rectal examination, the prostate often presents fixed nodules with increased consistency, and there may be a significant elevation of PSA in up to 40% of cases [2,3].

Multiparametric MRI (mpMRI) has become an important tool in the detection, localization, and characterization of lesions suggestive of prostate cancer (PCa), leading to the development of targeted prostate biopsy techniques. There are, however, benign conditions that mimic clinically significant PCa [4].

With this study, we intend to make an exposition of the cases of the Centro Hospitalar e Universitário de Lisboa Central (CHULC) regarding this theme, describing and evaluating the imaging findings, and exploring the history and clinical data of these patients.

## Materials and methods

For this study, all patients undergoing fusion prostate biopsy at CHULC in the period between January 2016 and December 2023 were taken into account. Their histopathological exams were analyzed, and those with GP were selected. Subsequently, the clinical histories of each of these patients were evaluated.

All mpMRIs were performed using a 1.5 Tesla MRI scanner (GE HealthCare, Chicago, IL, USA), following the European Society of Urogenital Radiology guidelines. The MRI protocol included T2-weighted imaging, diffusion-weighted imaging (DWI) with multiple b-values (including a high b-value of 1400 s/mm²), and dynamic contrast-enhanced (DCE) imaging. The interpretation of MRI images adhered to the Prostate Imaging Reporting and Data System (PI-RADS) guidelines, evolving from version 1 to version 2.1 over the course of the study [5].

Fusion-guided prostate biopsies were performed using a transperineal approach under general anesthesia. The biopsies were conducted using a bkFusion biopsy system (GE HealthCare, Chicago, IL, USA), which utilizes electromechanical fusion technology to integrate real-time ultrasound with MRI images. This allowed for precise targeting of suspicious lesions identified on mpMRI. Each biopsy session included both targeted biopsies of MRI-identified lesions and systematic biopsies to ensure comprehensive sampling of the prostate gland.

The biopsy samples were processed and stained with hematoxylin and eosin. A detailed histopathological examination was conducted by experienced pathologists to identify the presence of granulomas, multinucleated giant cells, necrosis, and other inflammatory cells characteristic of GP. Immunohistochemical staining, including CD68, was performed in selected cases to differentiate between GP and high-grade PCa. All histopathological findings were correlated with clinical and imaging data to confirm the diagnosis of GP.

## Results

Out of 1,232 patients, 12 showed lesions of GP. All were found to have a history of transurethral resection of high- or intermediate-risk non-muscle-invasive bladder urothelial carcinomas and subsequently underwent immunotherapy with BCG. All the data is summarized in Table 1.

**Table 1:** Patients' characteristics and MRI findings PSA: prostate-specific antigen, PI-RADS: Prostate Imaging Reporting and Data System, DWI: diffusion-weighted imaging, ADC: apparent diffusion coefficient, DCE: dynamic contrast enhanced

Patient	Age (years)	PSA (ng/mL)	Prostate volume (cc)	PSA density (ng/mL²)	Location	PI-RADS	Shape	Size (mm)	T2	DWI	ADC map	DCE
1	60	5.94	40	0.149	Peripheric zone	5	Pseudo-nodular	8	Hypointensity	Hypersignal	Hyposignal	No enhancement
2	64	9.75	144	0.068	Peripheric zone	4	Not defined	10	Intermidium Hypointensity	Hypersignal	Hyposignal	Focal enhancement
3	60	9.73	58	0.168	Peripheric zone	4	Nodular	10	Hypointensity	Hypersignal	Hyposignal	Focal enhancement
4	62	8.59	56	0.153	Transition zone	4	Non-circumscribed	8	Intermidium Hypointensity	Hypersignal	Hyposignal	No enhancement
5	46	7.26	35	0.207	Peripheric zone	4	Nodular	10	Hypointensity	Hypersignal	Hyposignal	Focal enhancement
6	61	4.95	66	0.075	Peripheric zone	5	Nodular	15	Hypointensity	Hypersignal	Hyposignal	Focal enhancement
7	70	7.32	75	0.098	Peripheric zone	5	Nodular	18	Hypointensity	Hypersignal	Hyposignal	Focal enhancement
8	65	4.60	35	0.131	Peripheric zone	4	Nodular	6	Hypointensity	Hypersignal	Hyposignal	Focal enhancement
9	72	5.78	64	0.090	Transition zone	4	Non-circumscribed	8	Hypointensity	Hypersignal	Hyposignal	No enhancement
10	59	9.54	55	0.173	Peripheric zone	5	Nodular	15	Hypointensity	Hypersignal	Hyposignal	Focal enhancement
11	68	7.18	92	0.078	Peripheric zone	4	Nodular	10	Intermidium hypointensity	Hypersignal	Hyposignal	No enhancement
12	75	11.20	110	0.102	Transition zone	4	Lenticular	10	Hypointensity	Hypersignal	Hyposignal	No enhancement

The median age of the patients was 62 years, with an interquartile range (IQR) of 60 to 68 years. The median PSA level was 7.26 ng/mL, with an IQR of 5.78 to 9.75 ng/mL. The median prostate volume was 58 cc, with an IQR of 40 to 75 cc. The median PSA density was 0.131 ng/mL², with an IQR of 0.078 to 0.168 ng/mL².

Of the 12 patients, eight experienced lower urinary tract symptoms, including both irritative and obstructive symptoms. On digital rectal examination, nodules were palpated in nine patients, indicating firm, irregular areas within the prostate, which raised initial concerns for malignancy.

**Figure 1:**
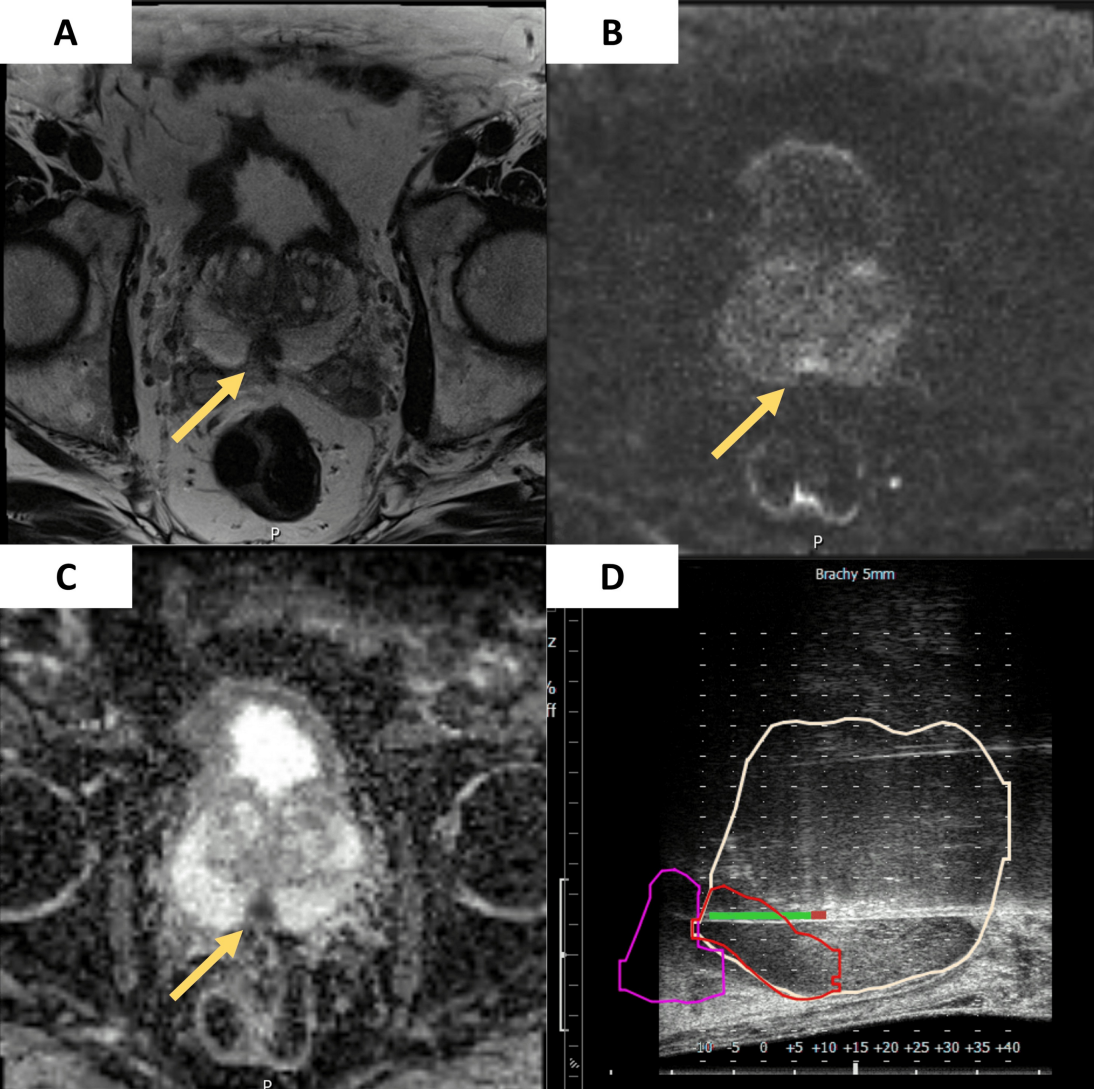
(A) Axial T2-weighted image with a nodular hypointense focal lesion (15 mm) in the peripheric zone (arrow). (B) On DWI with a high b value (1,400), a focal markedly hyperintense lesion (arrow). (C) With a markedly hypointense (arrow) value on the ADC map, consistent with a PI-RADS 5 lesion. (D) sagittal ultrasound view of the fusion-guided prostate biopsy, showing the prostate gland with the highlighted targeted lesion ADC: apparent diffusion coefficient, PI-RADS: Prostate Imaging Reporting and Data System, DWI: diffusion-weighted imaging

Most lesions (nine out of 12) were located in the peripheral zone, with the remaining three being in the transition zone. This distribution is significant as peripheral zone lesions are often associated with malignancies, posing a diagnostic challenge. High PI-RADS scores were observed, with four patients having PI-RADS five lesions and eight patients with PI-RADS four lesions. The majority of the lesions were nodular (eight out of 12), followed by non-circumscribed (two patients), pseudo-nodular (one patient), and lenticular (one patient). The median size of the lesions was 10 mm, with an IQR of 8 to 15 mm. Most lesions (10 out of 12) exhibited hypointensity on T2-weighted imaging. The remaining two lesions showed intermedium hypointensity. All 12 lesions displayed hypersignal on DWI and hyposignal on the ADC map, consistent with restricted diffusion and high cellularity (Figure 1). Focal enhancement was observed in seven patients, while no enhancement was seen in five patients.

**Figure 2:**
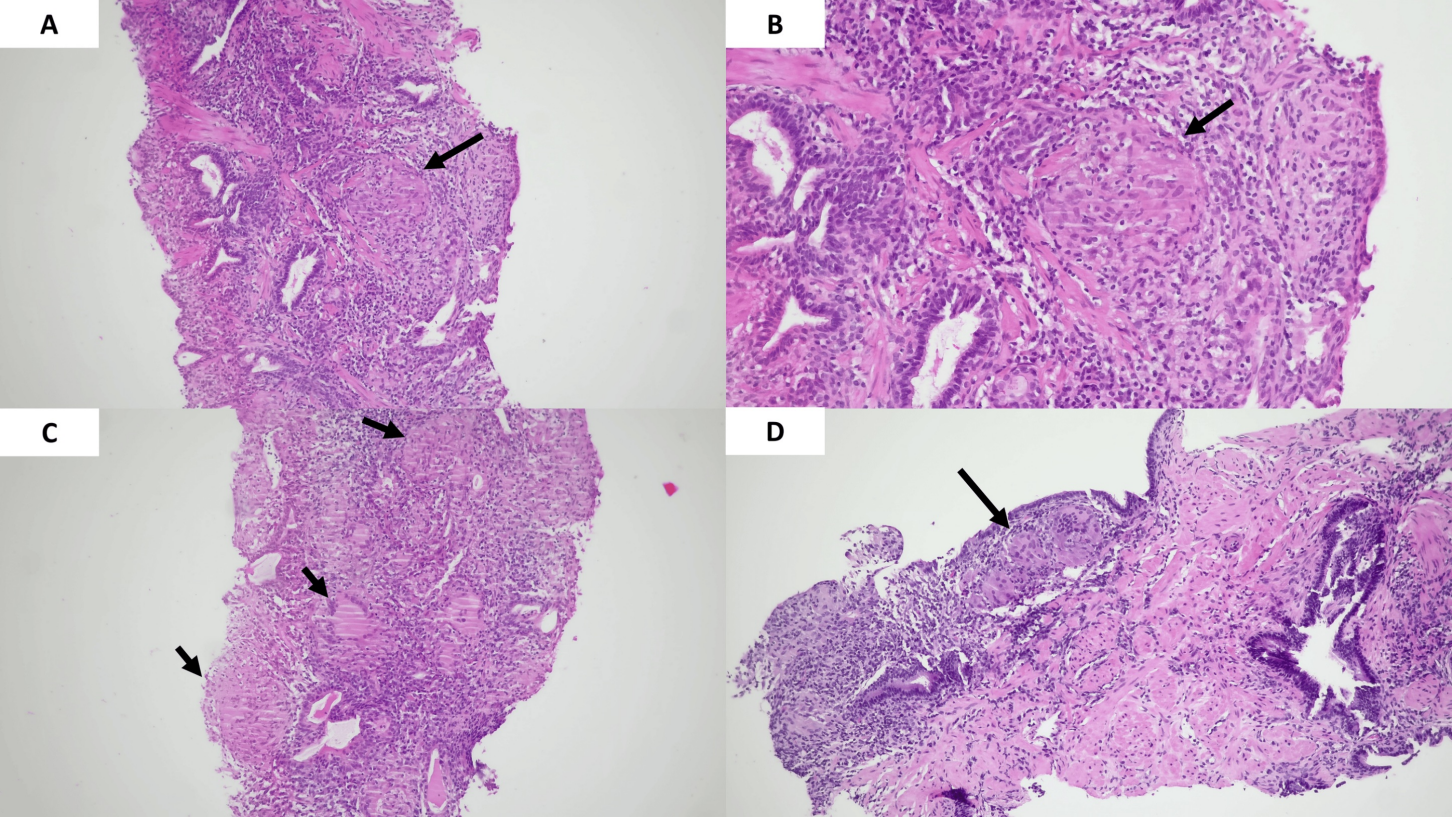
Histopathological examination of GP panels (A-D) show hematoxylin and eosin-stained sections of prostate tissue at different magnifications, highlighting features of GP. A (10x) and B (40x) display a granuloma with multinucleated giant cells and central necrosis (arrow). C (10x) shows multiple granulomas with peripheral palisading and surrounding mixed inflammatory infiltrate (arrows). D (10x) highlights a granuloma adjacent to benign prostatic tissue (arrow), showing the interface between inflamed and non-inflamed areas GP: granulomatous prostatitis

The histopathological examination typically reveals prostatic parenchyma with necrotizing epithelioid granulomas, which include multinucleated giant cells. This pattern is often surrounded by a mixed inflammatory infiltrate (Figure 2).

This detailed breakdown underscores the complexity of diagnosing GP, as its clinical and imaging features closely resemble those of PCa. The data highlights the necessity of histopathological confirmation to accurately diagnose this condition, reducing the risk of misdiagnosis and unnecessary treatments.

## Discussion

GP is an uncommon inflammatory condition of the prostate, first described by Tanner and McDonald in 1943. It accounts for approximately 3.3% of all benign inflammatory prostate lesions. Despite its benign nature, GP poses a significant diagnostic challenge due to its clinical and radiological similarities to PCa. The incidence of GP is expected to rise with the increasing use of intravesical BCG therapy for non-muscle-invasive bladder cancer [2,6].

It can be idiopathic or secondary to various causes, including infections, iatrogenic factors, and systemic diseases. BCG therapy is a significant etiological factor, responsible for up to 75% of GP cases in patients treated for non-muscle-invasive bladder cancer [3]. The pathogenesis involves the intraprostatic reflux of BCG-contaminated urine, leading to an intense localized inflammatory response characterized by granuloma formation [7].

Patients typically present with symptoms such as irritative and obstructive urinary complaints, including increased frequency, urgency, and dysuria. On digital rectal examination, the prostate often feels firm or nodular, similar to findings in PCa. PSA levels are usually elevated, which further complicates the differentiation from PCa. The elevated PSA can range from mild to significant increases, with levels reported between 0.88 and 50.9 ng/mL in various studies [6,8].

mpMRI plays a crucial role in the evaluation of prostatic lesions. GP lesions often mimic PCa, displaying hypointensity on T2-weighted images, high signal on DWI, and low ADC values. DCE MRI may show early and intense enhancement. These features are consistent across various studies, highlighting the challenge of distinguishing GP from malignant lesions based solely on imaging [9-12].

Our cases closely align with those previously described in the literature, particularly in terms of clinical presentation, imaging findings, and diagnostic challenges. The similarities reinforce the diagnostic difficulty in distinguishing GP from malignancy, emphasizing the importance of histopathological confirmation.

Histopathologically, it is characterized by the presence of granulomas - clusters of macrophages surrounded by mononuclear leukocytes and plasma cells. In some cases, foci of caseous necrosis may be present. The histopathological examination remains the gold standard for diagnosing GP. Immunohistochemical stains, such as CD68 for histiocytes, can aid in distinguishing GP from PCa, especially in florid cases where epithelioid histiocytes may mimic high-grade carcinoma [5,7,13].

The diagnostic dilemma posed by GP lies in its ability to closely mimic PCa in clinical, biochemical, and radiological aspects. While histopathology remains the gold standard for diagnosis, awareness of the GP's characteristic imaging features and clinical context can aid in raising suspicion for this condition, potentially reducing unnecessary biopsies. Advanced imaging techniques and criteria may further enhance diagnostic accuracy in the future. For example, AI-based analysis and the use of higher ADC values in the absence of high-stage features on mpMRI can help differentiate GP from PCa [2,6,14].

Management depends on the underlying cause. Idiopathic GP often resolves spontaneously and may only require symptomatic treatment. In cases secondary to infection, appropriate antimicrobial therapy is necessary. For GPs induced by BCG therapy, like all of our cases, management typically involves observation and symptomatic relief, as the condition often resolves on its own. However, in persistent or severe cases, anti-inflammatory medications or even surgical intervention may be required [13-15].

Our symptomatic patients were treated on alpha-blocker therapy, which was continued long-term, along with a two-week course of anti-inflammatory drugs. Following this treatment, six of the eight patients who had previously reported lower urinary tract symptoms experienced significant improvement, while two reported no noticeable change in their symptoms.

Despite the valuable insights provided by this study, several limitations must be acknowledged. First, the sample size of 12 patients is relatively small, which may limit the generalizability of the findings to a broader population. Including a control group of patients would provide a more thorough comparison. The single-center nature of the study further restricts the applicability of the results to other clinical settings. Additionally, while mpMRI was performed following standardized protocols, variations in image acquisition and interpretation could affect the reproducibility of the findings. The reliance on histopathological examination as the gold standard for diagnosing GP, while necessary, also highlights the challenge of differentiating GP from PCa based solely on imaging, emphasizing the need for further research with larger, multi-institutional cohorts to validate these findings.

## Conclusions

GP is a benign inflammatory condition of the prostate that can be difficult to distinguish from clinically significant PCa using clinical assessments and imaging alone. However, there are discrete features that can help guide the diagnosis. Even so, currently, the histopathological examination is crucial in their differentiation.

In the future, new techniques or the application of new criteria may emerge that may allow a better characterization of these lesions and, thus, possibly reduce the need for unnecessary biopsies.
